# Socioeconomic inequality in clusters of health-related behaviours in Europe: latent class analysis of a cross-sectional European survey

**DOI:** 10.1186/s12889-017-4440-3

**Published:** 2017-05-23

**Authors:** Shiho Kino, Eduardo Bernabé, Wael Sabbah

**Affiliations:** Division of Population and Patient Health, King’s College London Dental Institute at Guy’s, King’s College and St Thomas’ Hospitals, Bessemer Road, Denmark Hill, London, SE5 9RS UK

**Keywords:** Socioeconomic factors, Health behavior, Cluster analysis, Europe

## Abstract

**Background:**

Modifiable health-related behaviours tend to cluster among most vulnerable sectors of the population, particularly those at the bottom of the social hierarchy. This study aimed to identify the clusters of health-related behaviours in 27 European countries and to examine the socioeconomic inequalities in these clusters.

**Methods:**

Data were from Eurobarometer 72.3–2009, a cross-sectional survey of 27 European countries. The analyses were conducted in 2016. The main sections of the survey included questions pertaining to sociodemographic factors, health-related behaviours, and use of services. In this study, those aged 18 years and older were included. We selected five health-related behaviours, namely smoking, excessive alcohol consumption, frequent fresh fruit consumption, physical activity and dental check-ups. Socioeconomic position was indicated by education, subjective social status and difficulty in paying bills. Latent class analysis was conducted to explore the clusters of these five behaviours. Multinomial logistic regression model was used to examine the relationships between the clusters and socioeconomic positions adjusting for age, gender, marital status and urbanisation.

**Results:**

The eligible total population was 23,842. Latent class analysis identified three clusters; healthy, moderate and risky clusters in this European population. Individuals with the lowest socioeconomic position were more likely to have risky and moderate clusters than healthy cluster compared to those with the highest socioeconomic position.

**Conclusions:**

There were clear socioeconomic gradients in clusters of health-related behaviours. The findings highlight the importance of adopting interventions that address multiple health risk behaviours and policies that tackle the social determinants of health-related behaviours.

## Background

Modifiable health-related behaviours such as smoking, excessive alcohol consumption, poor diet, usually measured by fruit and vegetable consumption, and lack of physical activity have a major impact on a wide array of chronic conditions such as cancers, cardiovascular disease and diabetes, and on related mortality [1–3]. Interestingly, this set of risk behaviours is also correlated with oral health [4–7], which is related to the non-symptomatic use of dental services for check-ups [8, 9]. Furthermore, these five behaviours have common socioeconomic determinants that operate through similar pathways [10]. One phenomenon about these risk behaviours is that they tend to cluster among most vulnerable sectors of the population, particularly those at the bottom of the social hierarchy [11, 12]. Accumulation of negative life events and social disadvantage throughout the life also contributes to clustering of health risk behaviours [13]. However, there are a number of methodological challenges for clustering of health risk behaviours [14].

Given the cumulative and devastating impacts of the cluster of risk behaviours on health, there is a growing interest in research investigating this phenomenon to identify opportunities for effective interventions tackling multiple behaviours [14, 15]. Most of these studies have used co-occurrence of behaviours which is a count of risk or promoting behaviours [14]. This approach assumes homogeneity in the co-occurrence of behaviours within any given population. The utilisation of more complex analytical methods, such as latent class analysis, can help understanding the complex relationships among health-related behaviours. Such a technique utilises probability modelling to identify actual groups of behaviours within a cluster [16]. Earlier studies used latent class analysis for health-related behaviours [17, 18], however, none of them has estimated clusters of the five health-related behaviours used in this study.

Identifying socioeconomic determinants of different clusters of health-related behaviours is deemed important to enable selecting an effective approach to the population at risk. Most of the studies assessing socioeconomic inequalities in health-related behaviours used objective indicators such as income and education, which reflect material and educational pathways to health-related behaviours [14, 18, 19]. Although the difficulty in paying bills might not accurately reflect socioeconomic hierarchy as some individuals with higher income could have difficulty in paying bills, but it undoubtedly impacts ability to engage in healthy behaviours through materialistic pathway [20, 21]. Education is also linked to social position and it enables individuals to acquire knowledge related to enhancing health-related behaviours [22]. Using these common indicators that reflect material and educational pathways also allows comparison with other studies and between countries. On the other hand, these objective indicators of socioeconomic position do not completely capture the psychosocial pathway to inequality in behaviours which plays an important role in shaping health risk behaviours [23, 24]. For instance, the perception of relative deprivation is linked to stresses, insecurity, depression and social isolation which are all related to unhealthy behaviours [19, 24–27]. Using an indicator of subjective social status which reflects individuals’ perception of their social standing in their own country can capture the psychosocial pathway to inequality in health-related behaviours.

Occupation is widely used as a socioeconomic indicator, however the recent increase in low-level service jobs and decrease in manual jobs generate misleading classification of social hierarchy [28]. Furthermore, occupation classification does not capture unemployed, retired individuals, students and volunteer workers [28, 29]. In this study we used materialistic, educational and subjective indicators of socioeconomic position to test whether inequality exists in clusters of behaviours produced by latent class analysis in 27 European countries.

This study aims to identify different clusters of health-related behaviours using nationally representative samples in 27 European countries, and to examine the relationships between these clusters and objective and subjective indicators of socioeconomic position.

## Methods

### Data source and study sample

This study is a secondary analysis of cross-sectional survey in Europe, Eurobarometer 72.3, 2009. The survey included nationally representative samples from 27 European Union countries and three candidate countries [30]. A 2-stage, random (probability) sampling design was used for sample selection [30]. The data was collected by face-to-face interview in people’s home from October 2 to 19, 2009 by the TNS Opinion and Social through its network of national institutes in the respective national language. No more than one interview was conducted in each household [30].

The survey included data on 30,292 participants aged 15 years or older. The regular sample size was 1000 participants from each country with the exceptions of the United Kingdom (1000 for Great Britain and 300 for Northern Ireland), Germany (500 for the Eastern and 1000 for the Western) and Luxembourg, Cyprus Republic, Turkish Cypriot Community and Malta with 500 for each.

This study included participants who answered all questions pertaining to health-related behaviours and demographic/socioeconomic indicators. Given that smoking and drinking are illegal for those under 18 years in most of the European countries the analysis was limited to those aged 18 and over.

The survey included 26,013 participants aged 18 years or older in 27 European countries. After excluding those with missing values, 23,842 individuals aged 18 years or older in 27 European countries were included in this study (the valid percentage: 91.7%).

### Variables

#### Outcomes

The survey included questions about health-related behaviours in some domains, namely, check-up and medical screening, oral health, alcohol habits, smoking habits, and sport and physical activity. We selected five health-related behaviours, namely, smoking, excessive alcohol consumption, frequent fresh fruit consumption, physical activity which are highly correlated and linked to several non-communicable diseases, and oral diseases. Dental check-ups are also an important health-related behaviour that is linked to the aforementioned behaviours, highly related to socioeconomic indicators through the similar pathways as the other behaviours, and is not usually covered by the universal health coverage in Europe. These behaviours were dichotomised into binary options. Smoking was indicated by current smokers (versus former/never-smoker), which was based on self-reported with a single item. Excessive alcohol consumption was defined as having five or more drinks on one occasion at least once a week in the last 12 months. This is categorised based on the definition of risky single-occasion drinking as approximately 60-70 g ethanol for men and 40–60 g for women [31], which is equitable five standard drinks [31, 32]. Frequent fresh fruit consumption, as an indicator of healthy diet, included those who reported they often consume fruits versus those reporting “from time to time”, “rarely” or “never” for non-frequent fresh fruit consumption. A variable for physical activities was created by combining the two original questions, and was indicated by engaging in exercise, playing sports, or outdoor physical activity (e.g. cycling, walking from a place to another, dancing and gardening) at least four times a week, versus less than four times a week. European guideline indicated that physical activity at least five times a week is recommended for European adults [33]. As the dataset did not allow using this definition, in this study at least four times a week was used for physical activity as a cut-off point. Attendance for dental check-ups was indicated by use in the past 12 months either on own initiative, doctor’s initiative or in a screening programme.

#### Explanatory factors

The survey included four indicators of socioeconomic position, namely education, subjective social status, difficulty in paying bills and occupation. Given the limitations of occupational category, particularly when used in cross-country comparison, we opted to use the first three indicators which capture three different domains of socioeconomic position. Education was measured by the age when participants stopped full-time education, and was categorised into three groups: 20 years and older, 16–19 years old and 15 years or less. In most European countries, the minimum age for the compulsory education is 15 years or older, and the secondary school education is usually completed before 20 years of age [34]. Therefore, individuals in the lowest educational category were regarded as those who did not complete compulsory education, and those in the highest category were regarded as people entered a university level education. Participants still studying were included in the category corresponding to their age. Education is an important socioeconomic indicator as it is comparable across countries [35]. Subjective social status is appropriate for the comparison across countries as it reflects one’s perception of own status in the respective community/country. In the survey, participants were asked to place themselves on a ladder indicating their perception of own positions in their respective society on a scale of 1–10, hence reflecting perception of social standing. For better interpretation and to distinguish between the upper and the lower halves of the scale, subjective social status was categorised into quartiles, with the highest (step 7–10), the second highest (step 6), the second lowest (step 5) and the lowest (step 1–4). Difficulty in paying bills reflects the financial ability to pay bills at the end of the month during the last 12 months, and has three categories; most of the time, from time to time and almost never/never.

#### Demographic variables

Demographic factors included gender, age, urbanisation and marital status. Age was used as a continuous variable. Urbanisation has three categories (rural area or village, small or middle sized town and large town). Marital status was dichotomised to indicate; married/living with a partner, versus single/divorced/separated/widowed.

### Statistical analyses

#### Latent class analysis

To define which health-related behaviours naturally cluster together, latent class analysis was conducted using the five dichotomous variables by Mplus version 7.1. Latent class analysis has the most compelling methodological advantage in that it is based on probability modelling, which allows respondents to be assigned to the cluster to which they have the highest probability of belonging with taking into account that there is uncertainty about an object’s class membership [36].

A class-specific response probability, which is estimated in latent class analysis, indicates how likely it is that a participant belonging to a particular cluster (e.g. risky cluster) has a certain behaviour (e.g. smoking). In this study, we regarded a probability of 0.50 or lower as a low probability, a probability between 0.50 and 0.75 as moderate, and a probabilities of 0.75 or higher as high for interpretation [18].

#### Goodness-of-fit indexes

Latent class analysis does not automatically determine the number of clusters by one single measure. Hence, the goodness-of-fit indexes of the estimated models are used to select the most suitable model depending on the purpose with the following measures. The likelihood ratio-goodness-of-fit chi-squared statistic (*L* ^*2*^) indicates that the unexplained part of the observed relationships between the variables in the model. Hence, the smaller value indicates a model, which describes the better observed relationship and better fits the data. The *p*-value of *L* ^*2*^ is assessed based on the null hypothesis that the model is the true population model. The model fits data when *p*-value is more than 0.05 ideally [37].

There are some information criterions provided by latent class analysis, which weight model fit and parsimony. The smaller value indicates the better models [38]. The Bayesian Information Criterion (BIC) shows the value adjusting for the log likelihood value (*LL*) of the number of parameters (*Npar*) in the model. The adjusted BIC, which additionally adjusting for a sample size, correctly identifies the number of classes more consistently across all models and all sample sizes for categorical latent class analysis models in addition to that the BIC is superior to all other information criterions for all modelling settings [39]. In this study, as we conducted an exploratory latent class analysis in order to determine the best number of the clusters on the data, there were no restrictions to form the clusters. Therefore, we used BIC and adjusted BIC to determine the number of the clusters.

#### Assessment of association

To examine the relationships between socioeconomic indicators and clusters of health-related behaviours identified by latent class analysis, multinomial logistic regression model was used by Stata 12. The model included socioeconomic indicators (education, subjective social status and difficulty in paying bills) and demographic factors (age, gender, urbanisation and marital status), and the healthy cluster was used as a reference. Survey command and survey weights (the population sized and post-stratification weights) were used to account for the survey complexity and variations between countries and to produce population-level estimates.

## Results

A total of 23,842 participants from 27 European countries were included in this study after excluding those with missing data. As there were five health-related behaviours, models with one to four latent classes were estimated using latent class analysis. Model-fit indices are presented in Table 1. The one-cluster model was a baseline model, which assumes that the observed health-related behaviours are mutually independent and there is no association available to explain the relationship among the health-related behaviours. The model fit measures indicated that the three cluster model presented the most adequate solution for the data. Although the *p*-value corresponding to *L* ^*2*^ should formally be greater than 0.05 to conclude that a model fits the data, in this case it was considered acceptable given the very large sample size. Furthermore, the 3-cluster model had the lowest values BIC and adjusted BIC, which indicated that it was the preferred model according to that criterion.

**Table 1:** Model-fit indices for latent class analysis for health-related behaviours (*N* = 23,842; Eurobarometer 72.3, 2009, EU)

	*Npar* ^a^	*L* ^*2*b^	*df* ^c^	*L* ^*2*^ *p-value* ^d^	*LL* ^e^	BIC^f^	BIC*^g^
1-cluster model	5	1860.948	26	<0.001	−73,682.140	147,414.676	147,398.786
2-cluster model	11	264.915	20	<0.001	−72,884.123	145,879.117	145,844.160
3-cluster model	17	50.953	14	<0.001	−72,777.142	145,725.631	145,671.606
4-cluster model	23	19.064	8	0.0145	−72,761.198	145,754.217	145,681.124

^a^Number of parameters in the model

^b^Model Fit Likelihood ratio chi-squared statistic

^c^Degrees of freedom in the model

^d^ *p*-value of *L* ^*2*^

^e^Log likelihood

^f^Bayesian Information criterion, based on the log likelihood

^g^Bayesian Information criterion using sample size adjustment

**Fig. 1:**
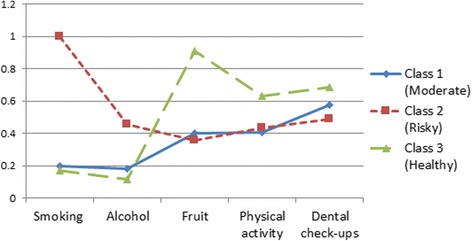
Cluster-specific probabilities of health-related behaviours for the three-cluster model (*N* = 23,842; Eurobarometer 72.3, 2009, EU)

Latent class analysis found three classes based on five health-related behaviours. The weighted percentages were 26.50% (*n* = 6312) for class 1, 15.84% (*n* = 3411) and 57.65% (*n* = 14,119). Figure 1 exhibits the cluster-specific estimated probabilities of health-related behaviours for the three-cluster model from latent class analysis. Class 1 had a moderate possibility of dental check-ups and lower possibilities for other four behaviours. Class 2 had a high probability of smoking and lower possibilities for other four variables. In addition, class 3 had a higher probability of fresh fruit consumption, and had a moderate probability of physical activity. Therefore, class 1 was named as “moderate cluster”, class 2 was named as “risky cluster” and class 3 was named as “healthy cluster”.

Table 2 shows the distribution of demographic indicators by the three clusters. Healthy cluster was more common among females, married individuals and those living in rural area/village and those with higher socioeconomic positions.

**Table 2:** Characteristics of the clusters (*N* = 23,842; Eurobarometer 72.3, 2009, EU)

	Healthy			Moderate			Risky			*P* value
	n	%	(95% CI)	n	%	(95% CI)	n	%	(95% CI)	
		Mean ± SE			Mean ± SE			Mean ± SE		
Gender										
Male	5132	40.25	(38.96, 41.56)	3143	55.00	(53.09, 56.89)	2178	64.48	(61.97, 66.92)	<0.001
Female	8987	59.75	(58.44, 61.04)	3169	45.00	(43.11, 46.91)	1233	35.52	(33.08, 38.03)	
Age in years	14119	50.40 ± 0.23		6312	46.30 ± 0.35		3411	40.25 ± 0.38		
Urbanisation										
Rural area/village	5162	35.19	(33.96, 36.44)	2286	35.17	(33.36, 37.03)	1176	32.68	(30.20, 35.27)	<0.001
Small/middle sized town	4991	40.49	(39.21, 41.79)	2203	39.65	(37.75, 41.58)	1166	38.37	(35.80, 41.01)	
Large town	3966	24.31	(23.25, 25.40)	1823	25.17	(23.59, 26.83)	1069	28.94	(26.65, 31.35)	
Marital status										
Married/with a partner	9534	68.00	(66.80, 69.18)	4052	65.04	(63.20, 66.84)	2022	58.88	(56.23, 61.48)	<0.001
Single	4585	32.00	(30.82, 33.20)	2260	34.86	(33.16, 36.80)	1389	41.12	(38.52, 43.77)	
Education										
20 years or older	4761	30.45	(29.28, 31.65)	1972	30.48	(28.71, 32.30)	841	24.26	(22.07, 26.58)	<0.001
16–19 years	6257	43.86	(42.58, 45.16)	2950	46.31	(44.39, 48.24)	1896	56.28	(53.64, 58.88)	
15 years or less	3101	25.68	(24.55, 26.85)	1390	23.22	(21.65, 24.86)	674	19.47	(17.52, 21.58)	
Subjective social status										
Highest	4347	28.85	(27.69, 30.03)	1741	25.61	(24.01, 27.29)	801	23.94	(21.77, 26.25)	<0.001
Second highest	3007	24.76	(23.63, 25.92)	1421	25.55	(23.86, 27.31)	663	20.44	(18.39, 22.66)	
Second lowest	4200	30.49	(29.30, 31.70)	1707	28.76	(27.01, 30.57)	1027	29.95	(27.51, 32.51)	
Lowest	2565	15.91	(15.01, 16.85)	1443	20.09	(18.61, 21.64)	920	25.67	(23.44, 28.03)	
Difficulty in paying bills										
Almost never/never	9669	70.66	(69.48, 71.82)	3948	64.66	(62.81, 66.47)	1675	52.3	(49.64, 54.94)	<0.001
From time to time	3419	23.55	(22.47, 24.66)	1759	27.08	(25.42, 28.82)	1173	34.15	(31.70, 36.69)	
Most of the time	1031	5.79	(5.25, 6.39)	605	8.25	(7.29, 9.34)	563	13.55	(11.94, 15.34)	

*P* value is presented from Chi-square test

There were clear socioeconomic gradients in clusters of health-related behaviours (Table 3). Individuals with the lowest socioeconomic positions were more likely to have risky or moderate cluster than healthy cluster compared with those with the highest socioeconomic position. Among the three socioeconomic indicators, difficulty in paying bills showed the most significant and consistent gradients in cluster of health-related behaviours with those unable to paying bills from time to time and most of the time having relative risk ratios of 1.58 (95% CI: 1.37, 1.83) and 2.44 (95% CI: 2.00, 2.99) for risky cluster compared to those without difficulty in paying bills. Females and older people were less likely to have moderate cluster and risky cluster than healthy cluster compared to males and younger people.

**Table 3:** Relative risk ratios for the relationships between socioeconomic indicators and clusters from multinomial logistic regression analyses (*N* = 23,842; Eurobarometer 72.3, 2009, EU)

	Moderate (vs Healthy)		Risky (vs Healthy)	
	RRR	(95% CI)	RRR	(95% CI)
Gender				
Male	1		1	
Female	0.53	(0.48, 0.59)	0.34	(0.30, 0.39)
Age in years	0.99	(0.98, 0.99)	0.97	(0.96, 0.97)
Urbanisation				
Rural area/village	1		1	
Small/middle sized town	0.99	(0.88, 1.10)	1.03	(0.89, 1.20)
Large town	0.99	(0.88, 1.12)	1.17	(0.99, 1.37)
Marital status				
Married/with a partner	1		1	
Single	1.16	(1.05, 1.29)	1.37	(1.20, 1.56)
Education				
20 years or older	1		1	
16–19 years	1.10	(0.98, 1.23)	1.74	(1.49, 2.03)
15 years or less	1.15	(0.99, 1.33)	1.73	(1.40, 2.13)
Subjective social status				
Highest	1		1	
Second highest	1.18	(1.04, 1.35)	0.99	(0.82, 1.19)
Second lowest	1.08	(0.95, 1.23)	1.15	(0.96, 1.38)
Lowest	1.37	(1.18, 1.59)	1.57	(1.29, 1.91)
Difficulty in paying bills				
Almost never/never	1		1	
From time to time	1.14	(1.02, 1.28)	1.58	(1.37, 1.83)
Most of the time	1.39	(1.16, 1.68)	2.44	(2.00, 2.99)

The model included age, gender, marital status, urbanisation, education, subjective social status, and difficulty in paying bills

## Discussion

This study firstly explored the clusters of five health-related behaviours, namely smoking, excessive alcohol consumption, fresh fruit consumption, physical activity and dental check-ups in the population in 27 European countries. Latent class analysis identified three clusters (healthy, moderate and risky clusters) in this population. Secondly, the study showed socioeconomic inequalities in the clusters of health-related behaviours, with those at the bottom of social hierarchy more likely to have risky and moderate clusters than those with the highest socioeconomic positions across all socioeconomic indicators.

This study used a latent class modelling to explore the clusters of health-related behaviours by the probability-based model rather than counting number of health risk behaviours in 27 European countries with nationally representative samples. Although it is also important to observe the number of health risk behaviours to reduce disease risks, the use of this clustering technique provides proper insights of the determinants of behaviours due to complexity of the relationships among health-related behaviours [16]. This study could potentially help to identify population at disease risk, which would possibly reduce the cumulative negative effects of health risk behaviours while improving efficiency [15].

Although a few other papers used latent class analysis, none of them examined socioeconomic inequality in the clusters [17, 18]. The current study uniquely examined five health-related behaviours including dental check-ups which reflect preventive use of services. This provides insight of the socioeconomic characteristics of clustering health-related behaviours from a different view in this specific population. The study demonstrated socioeconomic inequalities in this cluster of behaviours in a large sample of European.

One of the strengths of this study is the use of both objective and subjective socioeconomic indicators to examine socioeconomic inequalities. Education and difficulty in paying bills reflect educational/material inequality due to the nature of the indicators while subjective social status reflects subjective/psychosocial inequality because this indicator was measured by the comparison among the society where the individuals belong to. Education is associated with future jobs and income, and with improvement of healthy literacy and knowledge related to enhancing health-related behaviours [22]. In other words, highly educated individuals gave greater chances/opportunities to obtain and follow information pertaining to health-related behaviours than their least educated counterparts [20]. Although the allocation of people still studying might not reflect their socioeconomic position properly, it is assumed that the chances/opportunities for obtaining the information among people with the same age would be equally provided in the same educational categories. In addition, material ability is also correlated with participation in various health promoting behaviours. For example, the cumulative effect of economic hardship leads to poorer physical, psychological and cognitive functioning [40]. Thus, material deprivation affects health and health-related behaviours directly and indirectly. Subjective social status which largely reflect perception of status within own community is appropriate for cross-countries comparisons and is an important marker of relative deprivation which is linked to health and related behaviours through psychological pathways [41].

Difficulty in paying bills showed the highest odds ratios and the most consistent gradients in any models although education and subjective social status also showed significant gradients in the clusters of health-related behaviours. The results indicate that financial abilities had the strongest influence on having multiple health-related behaviours. Policies and strategies which support economically disadvantaged people could be more effective than behaviour changing intervention that tackles individual behaviours. Having said this, it is also worth noting that even better educated or employed individuals could perceive lower social status, and this may not be improved only with strategies of financial support. This observation suggests that developing and implementing specific interventions for socioeconomically disadvantaged individuals might not be sufficient to reduce inequalities in multiple health-related behaviours. On the other hand, a whole community approach that aims at improving the living environment for everyone might be more effective on producing sustainable changes in the behaviours of the whole society [42]. For example, in the past couple of decades banning smoking in many western countries appeared to be effective in reducing morbidity and mortality [43]. Similarly, introduction of safety measures for cyclists, availability of affordable healthy food, sugar and alcohol taxation could have a greater impact on the whole population than specific interventions targeting smaller groups of individuals [44, 45].

In addition, females were less likely, and singles were more likely to have risky cluster. Previous clustering studies using co-occurrence of number of health risk behaviours reported that more health risk behaviours were related to male gender and singles [11, 12]. This indicated that males and singles tended to have not only more number of risk behaviours but also a combination of health risk behaviours. The method used here helped identifying homogeneous clusters of behaviours and their socio-demographic determinants that could be generalised to European population.

This study has the advantage of using latent class analysis to identify healthy, moderate and risky clusters of five health-related behaviours including the four commonly used health-related behaviours and dental check-ups as an indicator for use of preventive services. The study also used three indicators of socioeconomic position reflecting different pathways to health-related behaviours in a large sample of the European countries.

There are a few limitations in this study. Firstly, this is a cross-sectional study, thus causality cannot be inferred. Secondly, some specific country’s characteristics, such as geographical, cultural, ethnic and macroeconomic factors, could have influenced the findings. However, the use of survey weights accounts for this limitation.

## Conclusion

There were clear socioeconomic gradients in these clusters of health-related behaviours with all socioeconomic indicators. The findings highlight the importance of adopting interventions that address multiple health risk behaviours and policies that tackle the social determinants of health-related behaviours.

## Abbereviations

BIC: Bayesian Information Criterion
*LL*: The log likelihood value
*L*^*2*^: The likelihood ratio-goodness-of-fit chi-squared statistic
*Npar*: The number of parameters
